# Synthesis and biological evaluation of esterified anti-inflammatory drugs with ethylene glycol linkers: cytotoxicity, anti-inflammatory and antioxidant properties

**DOI:** 10.1098/rsos.241413

**Published:** 2025-05-14

**Authors:** Pascaline M. Deussom, Monique Bassomo Ewonkem, Brice Enang, Michael H. K. Kamdem, Michel A. Mbock, Marthe C. D. Fotsing, Derek T. Ndinteh, Frederic N. Njayou, Flavien A. Toze

**Affiliations:** ^1^ Department of Chemistry, University of Douala, Douala, Cameroon; ^2^ Department of Biochemistry, University of Yaounde I Faculty of Sciences, Yaounde, Cameroon; ^3^ Department of Chemical Sciences, University of Johannesburg – Doornfontein Campus, Doornfontein, South Africa; ^4^ Department of Chemistry, University of Yaounde I Faculty of Sciences, Yaounde, Cameroon; ^5^ Department of Biochemistry, University of Douala Faculty of Sciences, Douala, Cameroon

**Keywords:** anti-inflammatory, antioxidant, cytotoxicity, esterification, multifunctional drugs, nitric oxide inhibition

## Abstract

The development of multifunctional drugs from anti-inflammatory agents is a promising strategy for people with several inflammation-related comorbidities since such medicines could reduce complications, improve health outcomes and lower healthcare costs. In this study, esters of ibuprofen, cinnamic and salicylic acids were synthesized and characterized by spectroscopic methods, with six new compounds identified. Cytotoxicity and anti-inflammatory properties were assessed using the 3-(4,5-dimethylthiazol-2-yl)-2,5-diphenyltetrazolium assay in mouse-derived peritoneal macrophages, which were obtained following an intraperitoneal injection of 0.5 ml of a 2% starch solution. All the tested compounds were safe up to 50% concentrations (2.41 × 10⁻⁴ to 2.41 mM), and monoethylene glycol di-ibuprofen (**2**) displayed the highest toxicity (IC_50_ = 4.90 mM). Most compounds were non-toxic below 2.41 mM, and all inhibited nitric oxide (NO) production in a concentration-dependent manner at 0.24 mM. Ibuprofen and cinnamic acid derivatives (**2**, **3**, **5a** and **14**) exhibited enhanced anti-inflammatory effects, with IC_50_ = 0.002 mM for monoethylene glycol mono-ibuprofen (**3**), while fatty-acid ester salicylates (**DEW4**) demonstrated weaker NO inhibition. Antioxidant tests (2,2-diphenyl-1-picrylhydrazyl, ferric reducing ability of plasma and 2,2′-azino-bis(3-ethylbenzothiazoline-6-sulfonate) (ABTS)) showed limited activities, with few compounds reducing the ABTS+ radical (0.1 ˂ SC_50_ ˂ 0.2 mM). Compounds **3**, **5a**, **7**, **12** and **14** are potential new anti-inflammatory drugs, while **2** may have anti-cancer properties.

## 1. Introduction

A significant portion of the elderly population suffers from multiple chronic illnesses—such as cardiovascular diseases, respiratory conditions, autoimmune disorders, diabetes, cancer and neurological disorders—where inflammation plays a critical role in both development and progression [1,2]. Patients often rely on multiple pharmaceutical agents for treatment, which can result in drug–drug interactions, drug intolerance and reduced clinical efficacy [3–7]. This underscores the urgent need to develop multifunctional drugs that are both effective and have lower toxicity, particularly those derived from anti-inflammatory agents, as inflammation is a key factor in these diseases [6,7].

Inflammation is a fundamental aspect of the immune system’s protective apparatus, facilitating the body’s ability to heal and repair [8]. However, if it persists or is not properly regulated, inflammatory responses can lead to the production of free radicals, which facilitate the elimination of pathogens and the removal of damaged tissue [9]. Though, excessive production of these radicals induces tissue damage and exacerbates the inflammatory response, provoking oxidative stress and the development of the above-mentioned diseases [10]. Furthermore, during the inflammatory process, immune cells (such as macrophages) produce nitric oxide (NO) in response to pro-inflammatory signals, thereby promoting the activity of immune cells and aiding in the destruction of pathogens [11,12]. At elevated concentrations, NO has been demonstrated to contribute to tissue damage and aggravate inflammatory conditions [13]. Its role in inflammation is particularly relevant in the context of various chronic diseases. For example, elevated levels of NO have been linked to neuro-inflammation and may play a role in the development of conditions such as Alzheimer’s disease [14].

Many studies have demonstrated that therapeutic compounds with anti-inflammatory properties like non-steroidal anti-inflammatory drugs (NSAIDs) and cinnamic acid derivatives have shown potential in treating diverse pathologies due to their antioxidant properties and low cytotoxicity [15–19]. These compounds can scavenge reactive oxygen species (ROS) and protect cellular structures, thereby mitigating oxidative stress and enhancing overall biological activity [20–24]. For example, acetylsalicylic acid (aspirin) is a NSAID with good antioxidant properties that contribute to beneficial cardiovascular effects, as it can reduce oxidative stress by scavenging OH radicals and protecting some cellular structures, including low-density lipoprotein (LDL), from oxidation [20,21]. Cinnamic acid derivatives have multiple biological activities, low cytotoxicity and antioxidant properties [18,19]. For example, 4-hydroxy-trans-cinnamic acid directly scavenges ROS by minimizing the oxidation of LDL [22,23]. Moreover, cinnamic acid has been shown to have the ability to enhance biological activity and reduce toxic side effects when incorporated into other compounds (e.g. natural products) via ester and amide linkages [19,25].

In fact, the presence of ester moieties in the chemical structure of a drug’s active ingredient has the advantage of increasing its lipophilicity, which is a crucial factor in determining its effectiveness as an antioxidant in lipophilic compartments and has a major impact on its ability to cross the cell membrane and reach its targets [26,27]. In addition, some studies demonstrated a strong relationship between cytotoxicity and lipophilicity of drugs. In fact, John *et al.* [28] found that moderate cytotoxicity of some drugs correlated with their relative lipophilicity, while most lipophilic drugs were also the most cytotoxic [28]. Moreover, Hizal *et al.* [24] evaluated by the 3-(4,5-dimethylthiazol-2-yl)-2,5-diphenyltetrazolium (MTT) assay the capacity of bis(carboxylato)oxalatobis(1-propylamine) platinum (IV) complexes and their ethylamine analogue to inhibit the proliferation of human cancer cell lines. They demonstrated that all compounds exhibited lower IC_50_ values that decreased with increasing lipophilicity within the compounds [24]. Also, the insertion or extension of alkyl groups into a molecule increases its lipophilicity [29].

Hence, synthesizing a single molecule with multiple therapeutic functions is becoming one of the key solutions to overcome the harmful effects of poly-medication. Knowing the correlation between lipophilicity and drug efficacy, this study focuses on the synthesis and chemical characterization (Fourier-transform infrared spectroscopy (FTIR), nuclear magnetic resonance (NMR) and high-resolution mass spectrometry (HRMS)) of multi-target esters of ibuprofen, cinnamic acid and salicylic acid using 1,2-ethylene glycol as a binder, followed by *in vitro* assessment of their cytotoxicity on macrophages and inhibition of NO production using the MTT assay, as well as their antioxidant capacity by the 2,2-diphenyl-1-picrylhydrazyl (DPPH), ferric reducing antioxidant power (FRAP) and 2,2′-azino-bis(3-ethylbenzothiazoline-6-sulfonate) (ABTS) tests.

## 2. Results and discussion

**Scheme 1.**
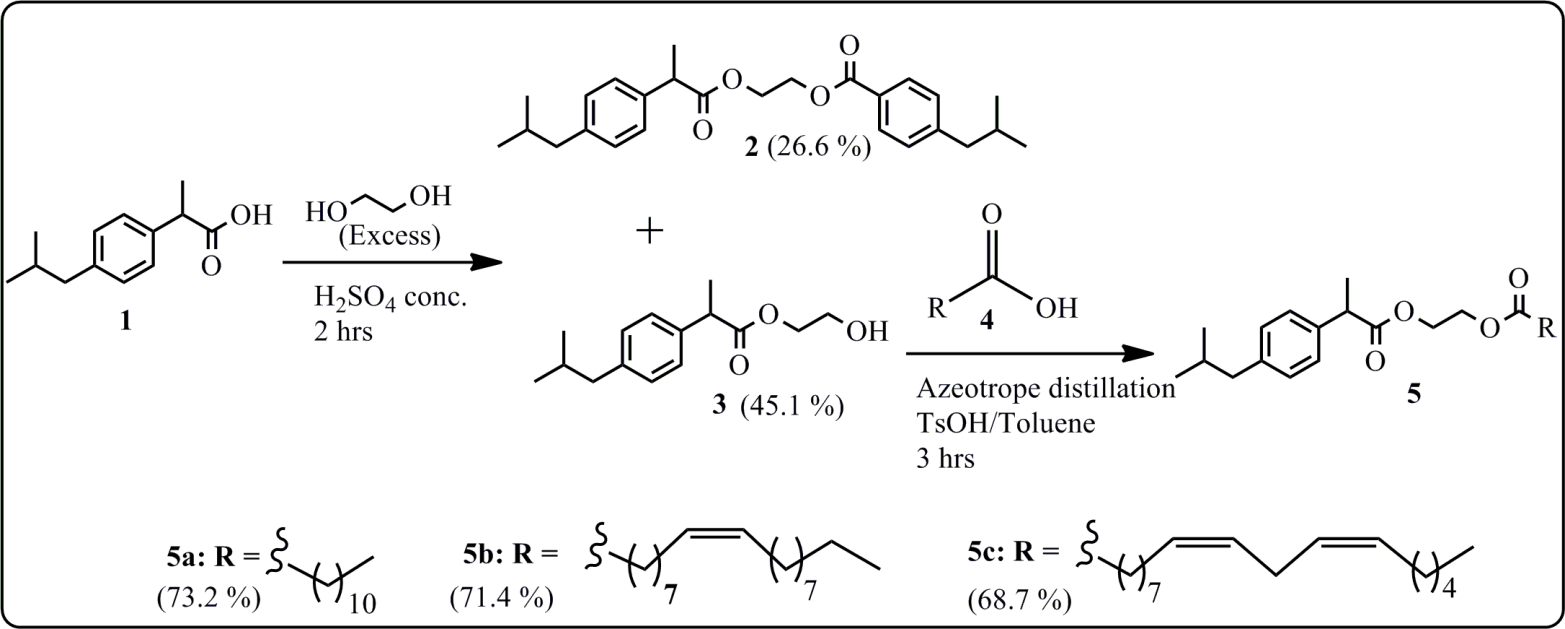
Synthesis of ibuprofen esters (**2**, **3**, **5**).

**Scheme 2.**
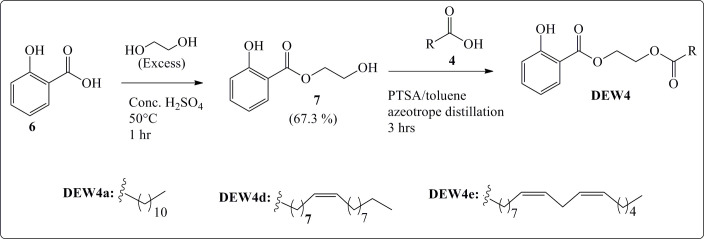
Synthesis of salicylate esters [21].

**Scheme 3.**
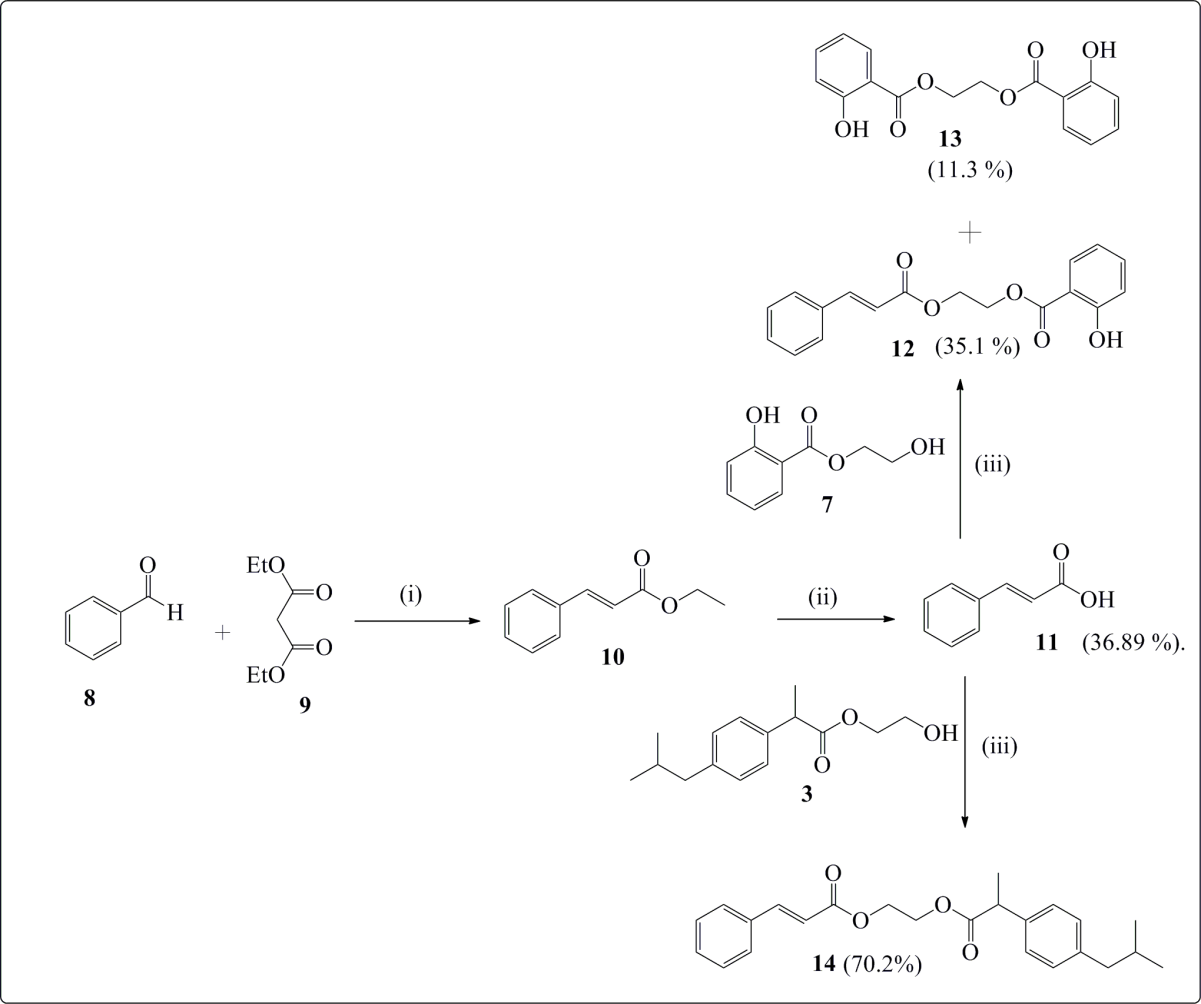
Synthesis of cinnamate esters (**13** and **14**). Reaction conditions: (i) piperidine/acetic acid/EtOH abs/reflux/2 h; (ii) KOH/EtOH abs./80 °C/reflux/2 h; (iii) azeotrope distillation/TsOH/toluene/3 h.

Three series of esters were synthesized from ibuprofen (scheme 1), salicylic acid (scheme 2) and cinnamic acid (scheme 3). Salicylic acid derivatives bearing fatty acid esters (**DEW4**) were previously synthesized [30], and their anti-inflammatory, antioxidant and cytotoxic properties were investigated here for the first time together with the new synthetic esters with a 1,2-ethylene glycol midsection obtained from the above-mentioned three starting materials.

### 2.1. Chemistry

#### 2.1.1. Synthesis of ibuprofen esters

Two groups of ibuprofen esters were synthesized (scheme 1), based on the esterification of ethylene glycol (**2**, **3**) and the esterification of ethylene glycol mono-ibuprofen with fatty acids (**5**).

##### 2.1.1.1. Synthesis of monoethylene glycol ibuprofens (**2–3**)

The monoethylene glycol ibuprofen derivatives (**2** and **3**) were synthesized by esterification of one mole of ibuprofen with an excess of absolute ethylene glycol under acidic conditions (scheme 1). These compounds are odourless and pale-yellow oily products obtained after purification of the reaction mixture by silica gel column chromatography. The analysis of the FTIR spectra provides evidence for the successful synthesis of compounds **2** and **3**. Indeed, it was observed a disappearance of the absorption band corresponding to the carbonyl group of the carboxylic acid present in ibuprofen (**1**) and the appearance of a new strong stretching band of the carbonyl at 1670 cm^−1^, indicative of ester formation, together with distinct peaks belonging to the benzene ring and aliphatic groups. A notable difference between the two compounds is highlighted by the presence of a large band of the OH group at 3300 cm^−1^ in compound **3**, which is clearly absent in compound **2**.

^1^H NMR spectra (see electronic supplementary material, figures S1 and S2) showed signals from both ibuprofen and ethylene glycol moieties. At lower magnetic fields, it was observed two signals of the four protons belonging to a para-disubstituted benzene ring (7.22−7.10 ppm). The four methylenic protons of the ethylene glycol unit appeared as a multiplet at 4.35 ppm in compound **2** and as two multiplets at 4.19 and 3.75 ppm in **3** for −CH_2_ deshielded by the carbonyl ester group and −CH_2_ bearing the primary alcohol group, respectively. Moreover, the spectrum of **2** allowed to prove its symmetry since the integration curves showed signals of eight aromatic protons together with one multiplet of the deshielded methylenic protons of the ethylene glycol fragment (electronic supplementary material, figure S1). All the aliphatic protons of the isobutyl and propionyl groups of ibuprofen were observed at higher magnetic fields from 2.50 to 0.90 ppm.

**Figure 1.**
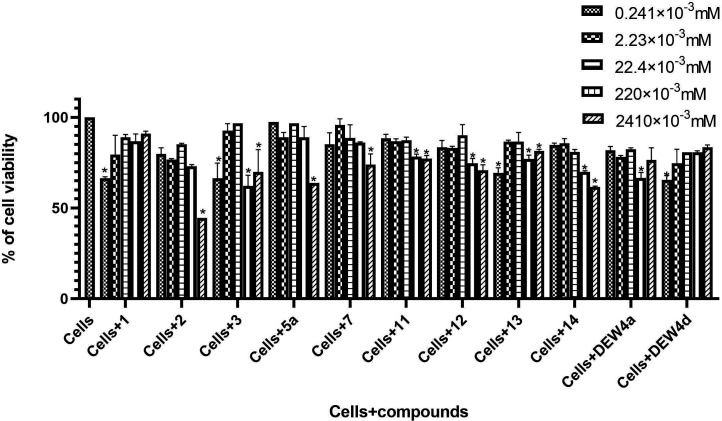
Effect of tested compounds on cell viability *significant difference at *p* ˂ 0.0001. **Cells =** inactivated cells (negative control).

**Figure 2.**
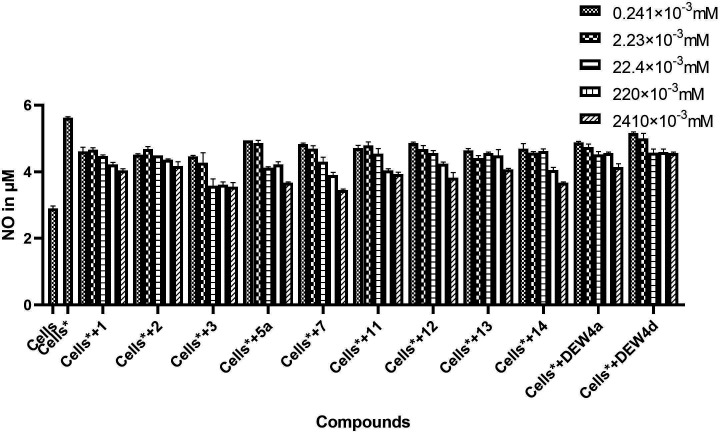
Inhibitory effect of tested compounds on nitric oxide production by macrophages. (Significances are presented on data of IC_50_ in table 1.) Cells* = cells activated with *Saccharomyces cerevisiae* (positive control) and **Cells** = inactivated cells (negative control).

**Table 1.** IC_50_ of NO inhibition assay. NO, nitric oxide.

samples	1	2	3	5a	7	11	12	13	14	DEW4a	DEW4d
IC_50_ (mM)	0.33	0.57	0.002	0.05	0.05	0.34	0.17	0.90	0.05	1.36	18.61**^a^**
s.d. (mM)	0.05	0.31	0.00	0.00	0.01	0.14	0.04	0.08	0.02	0.35	1.58

^a^ Significant difference at *p* ˂ 0.0001 compared with **1**, a known anti-inflammatory compound.

The ^13^C NMR spectra of ethylene glycol di-ibuprofen (**2**) and mono-ibuprofen (**3**) (electronic supplementary material, figures S3 and S4) showed the peaks of the carbons of the new carbonyl ester at 174.40 and 175.10 ppm, respectively. There was also a signal from methylenic carbons of the ethylene glycol moiety for compound **2** (62.24 ppm), confirming the symmetry of this molecule, and for **3**, there were two signals from the same fragment at 61.01 and 66.24 ppm (O = C-O-CH_2_-CH_2_-OH). The distortionless enhancement by polarization transfer (DEPT-135) spectra (electronic supplementary material, figures S5 and S6) showed downward signals from the CH_2_ groups, including two peaks for compound **2** and three for intermediate **3**.

The chemical structure of ibuprofen derivative **2** was confirmed by analysis of its HRMS (electronic supplementary material, figure S7) in a negative mode, where the pseudo molecular ion [M − H]^−^ was observed at *m/z* 437.2671 with the formula C_28_H_37_O_4_. Also in a negative mode, the peak of the pseudo molecular ion of derivative **3** [M − H]^−^ was seen at *m/z* 249.1479 together with a formula C_15_H_21_O_3_ (electronic supplementary material, figure S8).

##### 2.1.1.2. Synthesis of fatty acid ibuprofen esters (**5**)

Following the same procedure described in the literature [30], fatty acid ibuprofen esters were synthesized by reacting equimolar amounts of ethylene glycol mono-ibuprofen (**3**) and fatty acids (**4**) in toluene under azeotrope distillation for 3 h, in the presence of tosylic acid (TsOH) as catalyst. Fatty acids including lauric, oleic and linoleic acids were obtained by hydrolysis of palm kernel, olive and sunflower oils. The new aromatic fatty acid esters (**5**) are oily liquids at room temperature, and their chemical structures were confirmed by analysing their spectroscopic data (FTIR, NMR and HRMS).

FTIR spectra of the new ibuprofen derivatives (**5**) indicated the absence of the absorption bands of the OH and COOH groups. The presence of the stretching vibration peak of the carbonyl ester (1739 cm^−1^) was noted together with the C-H absorption bands of aliphatic and aromatic moieties.

The ^1^H NMR spectra of **5** (electronic supplementary material, figures S9–S11) displayed signals of ibuprofen, ethylene glycol and fatty acid moieties consisting of two doublets of the aromatic protons (7.22 and 7.11 ppm), a multiplet of the four methylenic protons vicinal to the oxygen atoms (4.27 ppm) and signals of the aliphatic protons (3.74–0.90 ppm) belonging to both the ibuprofen and fatty acid units (data from **5a**). Additionally, multiplets of the olefinic protons were observed for compounds **5b** and **5c** at 5.36 and 5.38 ppm, respectively.

^13^C NMR spectra of the fatty acid esters of ibuprofen (**5**) (electronic supplementary material, figures S12–S14) showed two peaks of the carbons of the carbonyl esters at 174.35 and 173.39 ppm, in addition to several carbon peaks of ibuprofen, ethylene glycol and fatty acid units.

The HRMS spectrum of the fatty acid ester of ibuprofen (**5a**) (electronic supplementary material, figure S15) indicated in a negative mode the peak of the pseudo molecular ion [M + Na − 2H]^−^ at *m/z* 453.2957, corresponding to the formula C_27_H_41_O_4_Na. The structures of **5b** and **5c** were also confirmed by analysis of their spectra (electronic supplementary material, figures S16 and S17) in a positive mode where pseudo molecular ions were observed at *m/z* 532.4166 ([M + H_2_O]^+^; C_33_H_55_O_5_) and *m/z* 535.3806 ([M+Na]^+^; C_33_H_52_O_4_Na), respectively.

### 2.1.2. Synthesis of salicylate esters

Following the procedure previously described by Ewonkem *et al.* [30], the salicylate esters (scheme 2) were obtained in a two-step esterification process from salicylic acid, an excess of ethylene glycol and fatty acids under hot and acidic conditions [30]. The chemical structures of the salicylate **7** and **DEW4a, d, e** were confirmed by comparison of their spectroscopic data (electronic supplementary material, figures S18–S29) with those previously synthesized.

### 2.1.3. Synthesis of cinnamate esters

Cinnamate esters synthesized here are cinnamate-ibuprofen (**12**) and cinnamate-salicylate (**13**) obtained by esterification of cinnamic acid with ethylene glycol mono-ibuprofen (**3**) and ethylene glycol mono-salicylate (**7**) (scheme 3).

Cinnamate esters of salicylate (**12**) and (**14**) ibuprofen were synthesized by reacting equimolar amounts of cinnamic acid (**11**) and monoethylene glycol derivatives (**3** or **7**) under azeotrope distillation in toluene with TsOH as catalyst for 3 h. The reaction was monitored by thin layer chromatography (TLC). After completion and usual work-up, pure compounds **12** (35.12%) and **13** (11.32%) were obtained by purification of the crude extracts of the reaction mixtures by silica gel column chromatography using *n*-hexane/ethyl acetate as eluent system.

However, the synthesis of cinnamate-salicylate (**12**) generated a by-product (**13**) which is a new compound appearing as light-pink powder. The FTIR spectrum of **13** showed a large absorption band at 3370 cm^−1^ for the phenolic OH group and a sharp elongation at 1671 cm^−1^ for the ester carbonyl. The ^1^H NMR spectrum of compound **13** (electronic supplementary material, figure S30) showed at lower magnetic fields an intense singlet of the chelated proton of the phenolic OH group at 10.62 ppm and signals of the aromatic protons (7.88−6.87 ppm). An intense singlet of methylenic protons belonging to the ethylene glycol moiety was seen at 4.73 ppm. Its DEPT-135 spectrum (electronic supplementary material, figure S31) confirmed that these protons were identical and also that the molecule was symmetrical, since only one peak of CH_2_ was seen in negative side at 62.71 ppm. The ^13^C NMR spectrum of **13** (electronic supplementary material, figure S32) showed among others the appearance of the peak of the new carbon of carbonyl ester at 169.9 ppm and the signal of the carbons of the CH_2_ group of the ethylene glycol unit. The chemical structure of compound **13** was confirmed by HRMS in a negative mode with the peak of pseudo molecular ion [M + Na − 2H]^−^ at *m/z* 323.0552 (electronic supplementary material, figure S33).

The cinnamate-salicylate (**12**) is a light-yellow powder with cinnamic and salicylic acid moieties separated by an ethylene glycol unit. Its ^1^HNMR spectrum (electronic supplementary material, figure S34) showed a singlet of the proton of the phenolic OH at 10.65 ppm, two doublets of the trans-olefinic protons at 7.72 and 6.47 ppm with *J*_trans_ = 16.0 Hz, seven signals between 6.89 and 7.88 ppm belonging to the aromatic protons of salicylate and cinnamate groups as well as two multiplets of the methylenic protons (O-CH_2_-CH_2_-O) at 4.66 and 4.59 ppm. ^13^C NMR spectrum (electronic supplementary material, figure S35) showed two peaks of the carbons of the ester carbonyl at 169.9 and 166.6 ppm for salicylate and cinnamate moieties, respectively. There were also signals of the sp^2^ carbon between 161.8 and 112.2 ppm, in addition to those of the two methylene groups vicinal to the oxygen atoms at 63.1 and 61.9 ppm. The success of the synthesis of **12** was completed by the analysis of its heteronuclear multiple bond correlation (HMBC) spectrum (electronic supplementary material, figure S36), where an important correlation was seen between the methylenic protons at 4.59 ppm of the ethylene glycol mono-salicylate unit and the carbon carbonyl of cinnamate (166.6 ppm), in addition to the correlation with carbon carbonyl of salicylate (169.9 ppm). The chemical structure of cinnamate-salicylate (**12**) was confirmed by analysing its HRMS spectrum (electronic supplementary material, figure S37) in a negative mode, where the pseudo molecular ion [M − H]^−^ was seen at *m/z* 311.0932, corresponding to the formula C_18_H_15_O_5_.

Finally, cinnamate-ibuprofen (**14**) is a yellow pasta with an ethylene glycol group linked at both ends by ibuprofen and cinnamate groups. Its ^1^H NMR spectrum (electronic supplementary material, figure S38) showed in lower magnetic fields two doublets of olefinic protons (7.69 and 6.36 ppm) characteristic of cinnamic acid moiety, four multiplets of nine aromatic protons (7.55−7.09 ppm). A multiplet of methylenic protons of the ethylene glycol linker at 4.39 ppm and aliphatic protons of ibuprofen from 3.76 ppm to 0.88 ppm were also observed. The DEPT-135 spectrum (electronic supplementary material, figure S39) exhibited in negative side three signals of CH_2_ carbons (62.3, 62.2 and 45.00 ppm), confirming the presence of ibuprofen ethylene glycol fragment and in positive side several peaks, among which the peaks of the methines of Csp^2^ (145.7 and 117.4 ppm) and Csp^3^ (30.08 and 45.03 ppm), together with the signals of methyl groups at 29.68 and 18.42 ppm. In the ^13^C NMR spectrum (electronic supplementary material, figure S40), it was noted the presence of two signals of carbonyl ester carbons at 174.51 ppm for ibuprofen unit and 166.55 for cinnamate fragment.

The formation of cinnamate-ibuprofen (**14**) was confirmed by analysis of the HRMS spectrum (electronic supplementary material, figure S41), in which the pseudo molecular ion in negative mode [M − H]^−^ was seen at *m/z* 379.1935, corresponding to the formula C_24_H_27_O_4_.

### 2.2. Biological evaluations

The biological activity of the substituted ibuprofen, cinnamic acid and salicylic acid products was evaluated in terms of their cytotoxicity on macrophages and inhibition of NO production, as well as their antioxidant capacity.

#### 2.2.1. Cytotoxicity and anti-inflammatory activity

The inflammatory response is part of the body’s first line of defence against a pathogen. Macrophages are one of the most studied inflammatory cells due to their high involvement in this inflammatory process [31]. The recognition of an antigenic motif by a toll-like receptor on macrophages leads to a cascade of molecular signalling to the synthesis of pro-inflammatory mediators such as NO to eliminate the pathogen [32]. First, the effect of the compounds on cell viability was assessed by measuring the cytotoxicity of the compounds in cell culture using the MTT assay at concentrations ranging from 2.41 × 10^–4^ to 2.41 mM (figure 1).

The various graphs (figure 1) showed that all the compounds have a proven cytotoxicity. However, they do not affect cell viability up to 50% in the range of concentrations used and do not allow their respective half cytotoxic concentration (IC_50_) to be clearly determined, which is greater than 2.41 mM. Monoethylene glycol di-ibuprofen (**2**) exhibited the highest toxicity among the tested compounds, with an IC_50_ = 4.90 ± 1.72 mM. This increased toxicity may be attributed to its greater lipophilicity, since it was demonstrated that the more lipophilic a compound is, the greater its cytotoxicity [28].

It was also found that below 2.41 mM, tested compounds are non-toxic to macrophages in culture and do not significantly affect cell viability. Therefore, for the most effective activity, tested compounds should be used at concentrations below 2.41 mM.

Second, the anti-inflammatory activity of these compounds at a maximum concentration of 0.22 mM was evaluated by assessing their ability to inhibit the synthesis of NO (figure 2).

NO and superoxide anion are highly synthesized when macrophages are stimulated by a specific antigen. Excessive accumulation of these two agents can lead to the formation of peroxynitrite (OONO-) and cause many inflammatory disorders. NO is also a vasodilator agent responsible for the excessive recruitment of inflammatory cells. Inhibition of any molecular target involved in the NO pathway of inflammation could have potential to inhibit inflammatory responses [33].

It was found that most of the tested compounds were able to significantly inhibit NO production in a concentration-dependent manner compared with the control (*p* ˂ 0.0001). According to the IC_50_ values, the anti-inflammatory activity of all the tested compounds, presented in table 1, is classified as follows: weak for IC_50_ ˃ 10 mM, moderate for 1 mM ˂ IC_50_ ˂ 10 mM and strong for IC_50_ ˂ 1 mM.

These results revealed that all the ibuprofen derivatives (**2**, **3**, **5a** and **14**) have strong anti-inflammatory activity, with compounds **3**, **5a** and **14** exhibiting superior anti-inflammatory properties (IC_50_ ≤ 0.05 mM) compared with ibuprofen (**1**) (IC_50_ = 0.33 mM) or monoethylene glycol di-ibuprofen (**2**) (IC_50_ = 0.57 mM). However, it was noticed that monoethylene glycol ibuprofen (**3**) is the most effective with a pronounced inhibitory activity (IC_50_ = 0.002 mM) probably because it possesses a primary alcohol group (OH) and only one ester moiety compared with the other derivatives that are diesters or with the ibuprofen that possesses the carboxylic acid group. Additionally, the decreasing anti-inflammatory activity of **2** could be due not only to the presence of two ester groups but also with the second ibuprofen unit that contributes to increase the lipophilicity of the molecule. A decrease in IC_50_ values is observed with the addition of one ibuprofen unit (aromatic ring), which enhances lipophilicity. This phenomenon is similarly evident when comparing monoethylene glycol (**7**) (IC_50_ = 0.05 mM) with its dimeric form (**13**) (IC_50_ = 0.90 mM). Furthermore, esterification of cinnamic acid (IC_50_ = 0.34 mM) with either monoethylene glycol salicylate (**7**) or monoethylene glycol mono-ibuprofen (**3**) yields compounds with enhanced anti-inflammatory, exemplified by compounds **12** (IC_50_ = 0.17 mM) and **14** (IC_50_ = 0.05 mM).

Moreover, ibuprofen derivatives exhibit superior anti-inflammatory activity compared with salicylates, probably due to their increased lipophilicity, which is reduced in salicylates by the phenolic OH group, impairing their biological interactions and anti-inflammatory efficacy. Finally, moderate to weak anti-inflammatory activities were observed for the diesters **13**, **DEW4a** and **DEW4d** (IC_50_ ˃ 1 mM) certainly because of the increasing chain length of the fatty acid moiety and the presence of the double bond carbon-carbon.

#### 2.2.2. Antioxidant properties

The *in vitro* antioxidant ability of the compounds synthesized in this work was evaluated using three different assays, including (i) the ABTS radical cation reduction-decolorization test, which uses the technique of Khan *et al.* [34] consisting of the reduction of the cation radical by an antioxidant through the discoloration measured by absorption at the wavelength of 734 nm; (ii) interaction with the stable free radical DPPH, based on Bassene’s method [35], in which the scavenging of DPPH radicals is carried out by a colorimetric method consisting of the reduction of the DPPH radical, determined by its absorption at a wavelength of 517 nm; and (iii) the FRAP test according to the protocol of Path Canada [36], based on the ability of a substance to reduce Fe^3+^ ions to Fe^2+^ ions, which, in the presence of 1,10-phenanthroline, form a red-orange complex whose optical density can be measured at 505 nm.

The antioxidant activity results of the synthesized compounds presented in table 2 are expressed as the concentration of radical scavengers required to neutralize 50% of the free radicals (SC_50_).

**Table 2.** Antioxidant activity of synthetic compounds (**1−3**, **5**, **7**, **DEW4**, **11−14**). ABTS, 2,2′-azino-bis(3-ethylbenzothiazoline-6-sulfonate); DPPH, 2,2-diphenyl-1-picrylhydrazyl, FRAP, ferric reducing ability of plasma. The bolded values indicate the compounds that exhibited superior antioxidant activity in reducing the ABTS⁺ cation radical, with concentrations ranging between 0.1 and 0.2 mM.

	ABTS				DPPH				FRAP			
samples	SC_50_(1) (mM)	SC_50_(2) (mM)	mean SC_50_ (mM)	s.d.	SC_50_(1) (mM)	SC_50_(2) (mM)	mean SC_50_ (mM)	s.d.	SC_50_(1) (mM)	SC_50_(2) (mM)	mean SC_50_ (mM)	s.d.
**1**	**0.21**	**0.20**	**0.20**	**0.003**	9.81	10.18	10.00	ND	12.89	13.21	13.05	ND
**2**	1.67	1.73	1.70	ND	5.05	4.78	4.92	ND	6.79	6.79	6.79	ND
**3**	3.70	4.11	3.90	ND	10.47	10.39	10.43	ND	7.58	7.39	7.48	ND
**5a**	2.77	2.81	2.79	ND	3.50	3.53	3.52	ND	4.66	4.67	4.67	ND
**5b**	2.15	2.11	2.13	ND	6.44	6.52	6.48	ND	6.16	6.20	6.18	ND
**7**	**0.21**	**0.21**	**0.21**	**0.00**	12.20	12.06	12.13	ND	10.97	10.88	10.92	ND
**11**	5.83	5.98	5.91	ND	11.10	10.95	11.02	ND	13.83	13.64	13.73	ND
**12**	3.36	3.60	3.47	ND	8.63	8.73	8.68	ND	6.21	6.22	6.21	ND
**13**	**0.21**	**0.20**	**0.20**	**0.007**	6.61	6.46	6.47	ND	6.73	6.66	6.69	ND
**14**	**0.18**	**0.18**	**0.18**	**0.005**	6.64	6.68	6.13	ND	7.68	7.27	7.75	ND
**DEW4a**	3.24	3.30	3.25	ND	5.82	5.77	5.79	ND	8.06	8.00	8.03	ND
**DEW4d**	**0.16**	**0.17**	**0.16**	**0.007**	8.18	7.89	8.03	ND	4.50	4.53	4.51	ND
**gallic acid**	0.038	0.038	0.035	0.000	0.015	0.015	0.015	0.000	0.059	0.062	0.061	0

>1: not active at tested concentration.

ND: not determined

It was observed that all the compounds tested are unable to scavenge free radicals H and Fe since their SC_50_ are higher than 1 mM in the DPPH and FRAP tests. However, five of them showed antioxidant capacity by reducing ABTS+ cation radical with SC_50_ values between 0.1 and 0.2 mM. The best score being made by the cinnamic acid (SC_50_ = 0.21 mM).

## 3. Experimental

### 3.1. Materials

Reagents like benzaldehyde (≥99%), diethyl malonate (99%), salicylic acid (≥99.0%), ethylene glycol (anhydrous, 99.8%), tosylic acid (≥98.5%), piperidine (99%), N,N'-dicyclohexylcarbodiimide (DCC) (99%), N,N-diméthyl-4-aminopyridine (≥99%) and ibuprofen (≥98%) were purchased from Sigma-Aldrich (Germany). Vegetable oils were bought from Cameroonian markets: palm kernel oil (Nona), olive oil (Puget) and sunflower oil (Excella). Solvents such as methylene chloride (≥99.5%, VWR Chemicals BDH^®^), toluene (≥99.5%, Fisher Chemical™), ethanol (95%, Fisher Chemical), ethyl acetate (99%, Thermo Scientific Chemicals) and *n*-hexane (≥95%, Thermo Scientific Chemicals) were supplied by PROMACAM company in Cameroon.

FTIR analyses were carried out with a SHIMADZU Fourier Transformation InfraRed Spectrophotometer IRSpirit-T with a single-reflection attenuated total reflectance (ATR) accessory QATR-S. NMR analysis was carried out in CDCl_3_ and DMSO-d_6_ solvents, with a magnet of 500 MHz coupled to an Avance III HD 500 MHz console. All spectra were referenced using the chemical shifts of the different solvents, both for ^1^H and ^13^C. Tetramethylsilane was used as the internal standard. High-resolution mass spectra were obtained using a compact quadrupole time of flight spectrometer (Bruker, Germany) equipped with a high‐resolution electrospray ionization (HRESI) source. The spectrometer was operated in a negative mode (mass range: 50–1500, with a scan rate of 1.00 Hz) with automatic gain control to provide high-precision mass measurements within 0.4 ppm deviation using Na formate as the calibration agent with nitrogen as the sheath gas (4 l min^−1^), a spray voltage of 4.5 kV and a capillary temperature of 200°C.

### 3.2. Methods

#### 3.2.1. Synthesis

##### 3.2.1.1. Synthesis ibuprofen esters

###### 3.2.1.1.1. Synthesis of ibuprofen-ethylene glycol (**2** and **3**)

A total of 15 g of ibuprofen (73 mmol), 60 ml of ethylene glycol (727 mmol) and 4.5 ml of concentrated H_2_SO_4_ (96 mmol) were refluxed at 80°C for 2 h. The reaction was monitored by TLC using the eluent system *n*-hexane/ethyl acetate (3/1, v/v), comparing samples of the two starting materials with very small amounts of the reaction mixture taken every half hour. The reaction was complete when ibuprofen, the standard reagent, was completely consumed. After completion, the reaction mixture was cooled down at room temperature and dissolved in distilled H_2_O. Then, the organic phase was washed with an aqueous solution of NaHCO_3_ (5%) and three times with distilled H_2_O till neutral pH. The organic phase was collected, dried with anhydrous MgSO_4_, filtered and evaporated under vacuum. The crude extract was purified by silica gel column chromatography using a mixture of solvents with increasing polarity gradient (*n*-hexane/ethyl acetate: 5/1 and 2/1, v/v) to yield ibuprofen mono ethylene glycol (**3**) and di-ibuprofen mono glycol ethylene glycol (**2**) that were characterized by common spectroscopic methods (FTIR, NMR and HRMS-ESI).

*Di-ibuprofen mono ethylene glycol (* ***2*** *): 2-((2-(4-isobutylphenyl)propanoyl)oxy)ethyl 4-isobutylbenzoate*

Pale yellow oil (7.81 g, 26.6%). FTIR (υ_max_ cm^−1^): 1734 (conj. C=O), 2955(C-H aliphatic), 1246 (O=C–O–C). *δ*_H_ (500 MHz, CDCl_3_, Me4Si) : 7.20 (4H, d, *J* = 7.8 Hz, Ph), 7.11 (4H, d, *J* = 7.9 Hz, Ph), 4.26 (4H, m, O-C*H*_2_-CH_2_-O), 3.68 (2H, dt, *J* = 7.2 & 3.6 Hz, C*H*-C=O), 2.47 (4H, d, *J* = 7.1 Hz, C*H*_2_-CH(CH_3_)_2_), 1.88 (2H, m, (CH_3_)_2_C*H*-CH_2_), 1.48 (6H, d, *J* = 7.1 Hz, C*H*_3_-CH), 0.93 (12H, dd, *J* = 6.6 Hz, (C*H*_3_)CH). *δ*_C_ (125 MHz, CDCl_3_, Me4Si): 174.4, 140.5, 137.5, 129.3, 127.1, 62.2, 45.0, 30.1, 22.3, 18.4. HRMS-ESI in a negative mode (*m/z*): calcd for C_28_H_38_O_4_ [M]^−^ = 438.2776; C_28_H_37_O_4_ [M−H]^−^ = 437.2697. Found [M−H]^−^ = 437.2671.

*Ibuprofen mono ethylene glycol (* ***3*** *): 2-hydroxyethyl 2-(4-isobutylphenyl)propanoate*

Pale yellow oil (6.73 g, 45.1%). FTIR (υ_max_ cm^−1^): 3215 (OH), 2955 (C-H), 1734 (conj. C=O). *δ*_H_ (500 MHz, CDCl_3_, Me4Si) : 7.22 (2H, d, *J* = 8.1 Hz, Ph), 7.12 (2H, d, *J* = 8.1 Hz, Ph), 4.20 (2H, m, OCO-C*H*_2_), 3.75 (2H, m, -C*H*_2_-OH), 3.74 (1H, m, -C*H*-C=O), 2.47 (2H, d, *J* = 7.2 Hz, C*H*_2_-CH(CH_3_)_2_), 1.87 (1H, m, (CH_3_)_2_C*H*-CH_2_), 1.52 (3H, d, *J* = 7.2 Hz, C*H*_3_-CH), 0.92 (6H, dd, *J* = 6.7 Hz, C*H*_3_-CH). *δ*_C_ (125 MHz, CDCl_3_, Me4Si): 175.1, 140.6, 137.6, 129.4, 127.0, 66.2, 61.0, 45.0, 30.1, 22.3, 18.4. HRMS-ESI in a negative mode (*m/z*): calcd for C_15_H_22_O_3_ [M]^-^ = 250.1574; C_15_H_21_O_3_ [M−H]^−^ = 249.1496. Found [M−H]^−^ = 249.1479.

### 3.2.1.2. Synthesis of fatty acid-ibuprofen esters (**5**)

#### 3.2.1.2.1. Synthesis of fatty acids

Lauric, oleic and linoleic acids were, respectively, synthesized by hydrolysis of palm kernel, olive and sunflower oil, according to the procedure described in the literature [30]. *General procedure*: 20.0 g of vegetable oil, 3.95 g of KOH (71 mmol) in 48 ml of abs. EtOH (820 mmol) were refluxed for 2 h. TLC, using *n*-hexane/ethyl acetate (5/1, v/v) as eluent, monitored the reaction. After neutralizing the reaction mixtures and washing several times the organic phases, pure fatty acids were obtained and characterized by FTIR, NMR and HRMS-ESI.

### 3.2.1.3. Synthesis of ibuprofen fatty acids (**5**)

Fatty acid ibuprofen esters (**5**) were obtained following the chemical procedure described in the literature [30]. Equimolar amounts of ethylene glycol mono-ibuprofen (**3**) and fatty acids (**4**) in toluene were heated under azeotrope distillation for 3 h, in the presence of TsOH as a catalyst. Pure compounds were obtained by purification using silica gel column chromatography (*n*-hexane/acetate: 10/1, v/v), and their structures were confirmed by analyses of their spectroscopic data including FTIR, NMR and HRMS-ESI spectra.

*Laurate ethylene glycol ibuprofen (**5a***): *2-((2-(4-isobutylphenyl)propanoyl)oxy)ethyl dodecanoate*

Oily transparent liquid (2.53 g, 73.2%). FTIR (υ_max_ cm^−1^): 2954 (aliphatic C-H), 1738 (C=O ester), 1153 (C-O). *δ*_H_ (500 MHz, CDCl_3_, Me4Si) : 7.22 (2H, d, *J* = 8.0 Hz, Ph), 7.11 (2H, d, *J* = 8.0 Hz, Ph); 4.27 (4H, m, O-C*H*_2_-C*H*_2_-O), 3.74 (1H, q, *J* = 6.5 Hz, C*H*-C=O), 2.47 (2H, d, *J* = 7.2 Hz, C*H*_2_-CH(CH_3_)_2_), 2.27 (2H, t, *J* = 7.6 Hz, CH_2_-C*H*_2_-COO), 1.87 (1H, m, (CH_3_)_2_C*H*-CH_2_), 1.61 (2H, m, C*H*_2_-), 1.52 (3H, d, *J* = 7.2 Hz, C*H*_3_-CH-COO), 1.29 (16H, m, C*H*_2_), 0.92 (6H, d, *J* = 6.7 Hz, (C*H*_3_)_2_-CH), 0.91(3H, t, *J* = 7.0 Hz, C*H*_3_-CH_2_). δ_C_ (125 MHz, CDCl_3_, Me4Si): 174.4, 173.4, 140.5, 137.5, 129.2, 127.1, 62.3, 61.8, 45.0, 34.0, 31.9, 30.1, 29.6, 29.5, 29.3, 29.3, 29.1, 24.8, 22.7, 22.4, 18.4, 14.1. HR-MS-ESI in negative mode (*m/z*): calcd for C_27_H_44_O_4_ [M]^-^ = 432.3245; C_27_H_42_O_4_Na [M + Na − 2H]^−^ = 453.2986. Found [M + Na − 2H]^−^ = 453.2957.

*Oleate ethylene glycol ibuprofen (* ***5b*** *): 2-((2-(4-isobutylphenyl)propanoyl)oxy)ethyl oleate*

Oily transparent liquid (2.93 g, 71.4%). FTIR (υ_max_ cm^−1^): 2923 & 2853 (C-H), 1740 & 1676 (C = O ester), 1615 (C = C), 1157 (C-O). *δ*_H_ (500 MHz, CDCl_3_, Me4Si) : 7.22 (2H, d, *J* = 8.0 Hz, Ph), 7.11 (2H, d, *J* = 8.0 Hz, Ph), 5.38 (2H, m, C*H* = C*H*), 4.28 (4H, m, O-C*H*_2_-C*H*_2_-O), 3.74 (1H, q, *J* = 7.1 Hz, C*H*-C=O), 2.46 (2H, d, *J* = 7.2 Hz, C*H*_2_-CH(CH_3_)_2_), 2.34 (2H, t, *J* = 7.5 Hz, CH_2_C*H*_2_-COO), 2.27 (4H, m, CH=CH-C*H*_2_), 1.86 (1H, m, (CH_3_)_2_C*H*-CH_2_), 1.51 (3H, d, *J* = 7.2 Hz, C*H*_3_-CH-COO), 1.30 (16H, m, C*H*_2_), 0.92 (6H, d, *J* = 6.9 Hz, (C*H*_3_)_2_-CH), 0.90 (3H, t, *J* = 7.5 Hz, C*H*_3_-CH_2_,). δ_C_ (125 MHz, CDCl_3_, Me4Si): 174.5, 173.5, 140.6, 137.5, 129.3, 127.2, 62.4, 61.8, 45.1, 34.1, 31.9, 30.2, 29.7, 29.3, 27.2, 24.8, 22.4, 18.5, 14.1. HR-MS-ESI in a positive mode (*m/z*): calcd for C_33_H_54_O_4_ [M]^+^ = 514.4028; C_33_H_55_O_5_ [M + H_2_O]^+^ = 532.4133. Found [M + H_2_O]^+^ = 532.4166.

### 3.2.1.4. Linoleate ethylene glycol ibuprofen (**5c**): (9Z,12Z)-2-((2-(4-isobutylphenyl)propanoyl)oxy)ethyl octadeca-9,12-dienoate

Oily transparent liquid (2.73 g, 68.7%). FTIR (υ_max_ cm^−1^): 2923, 2853(C-H), 1740, 1679 (C = O ester), 1615 (C=C), 1156 (C-O). *δ*_H_ (500 MHz, CDCl_3_, Me4Si) : 7.19 (2H, d, *J* = 8.1 Hz, Ph), 7.08 (2H, d, *J* = 8.1 Hz, Ph), 5.36 (4H, m, C*H*=C*H*), 4.25 (4H, m, O-C*H*_2_-C*H*_2_-O), 3.71 (1H, q, *J* = 7.2 Hz, C*H*-C=O), 2.44 (2H, d, *J* = 7.2 Hz, C*H*_2_-CH(CH_3_)_2_), 2.32 (2H, t, *J* = 7.6 Hz, CH_2_C*H*_2_-COO), 2.24 (8H, m, CH=CH-C*H*_2_), 1.83 (2H, m, (CH_3_)_2_C*H*-CH_2_), 1.52 (2H, d, *J* = 7.5 Hz, C*H*_3_-C*H*-COO), 1.49 (3H, d, *J* = 7.0 Hz, C*H*_2_), 1.26 (14H, m, CH_2_), 0.91 (6H, d, *J* = 6.5 Hz, (C*H*_3_)_2_-CH), 0.89 (3H, t, *J* = 7.5 Hz, C*H*_3_-CH_2_). δ_C_ (125 MHz, CDCl_3_, Me4Si): 174.5, 173.5, 140.6, 137.5, 131.0, 129.3, 127.2, 62.4, 61.8, 45.0, 34.1, 30.2, 29.7, 29.1, 27.2, 24.8, 22.7, 18.5, 14.1. HR-MS-ESI in a positive mode (*m/z*): calcd for C_33_H_52_O_4_ [M]^+^ = 512.3871; [M + Na]^−^ = 535.3769. Found [M + Na]^+^ = 535.3806.

### 3.2.2. Synthesis of cinnamic esters

#### 3.2.2.1. Synthesis of cinnamic acid (**9**)

A mixture of 10 g of benzaldehyde (94 mmol) and 18.28 g of diethyl malonate (1141 mmol) together with two drops of acetic acid and 1 g of piperidine (117 mmol) in absolute ethanol (54.73 ml, 940 mmol) was refluxed for 2 h. The reaction mixture was monitored by TLC (*n*-hexane/ethyl acetate: 5/1, v/v). At the end of the reaction, the solvent was evaporated, and the residue was dissolved in distilled H_2_O. Ethyl acetate was added to the aqueous solution. The organic phase was washed twice with distilled H_2_O, collected, dried with anhydrous MgSO_4_ then evaporated to give the cinnamate ethyl ester. An ethanolic KOH solution was added to the cinnamate ethyl ester and refluxed for 1 h. The reaction mixture was evaporated, distilled H_2_O was added and the aqueous solution was acidified till pH = 1. Then, ethyl acetate was added to the obtained solution, the organic phase was washed several times with distilled H_2_O until a neutral pH was reached. The organic phase was collected, dried with anhydrous MgSO_4_, and the solvent was evaporated to yield a brown extract, which was purified by silica gel column chromatography using hexane/ethyl acetate (4/1 and 2/1, v/v) to give cinnamic acid (36.89%) as a pure, which was characterized by FTIR, NMR and comparative spectroscopic data found in the literature [37].

White powder (5.15 g, 36.89%). FTIR (υ_max_ cm^−1^): 2805 (C-H), 1685 (conj. C=O), 1629 (C=C). δ_H_ (500 MHz, CDCl_3_, Me4Si): 8.14 (1H, d, *J* = 7.5 Hz, Ph), 7.83 (1H, d, *J*_trans_ = 16.0 Hz, C*H* = C*H*), 7.56 (2H, *J* = 6.7 & 2.9 Hz, Ph), 7.48 (2H, dd, *J* = 5.0 & 1.9 Hz, Ph), 6.47 (1H, d, *J*_trans_ = 16.0 Hz, C*H*=C*H*) (electronic supplementary material, figure S30). δ_C_ (125 MHz, CDCl_3_, Me4Si): 172.3, 147.1, 134.1, 133.7, 130.7, 130.2, 129.0, 128.4, 117.3 (electronic supplementary material, figure S31).

#### 3.2.2.2. Monoethylene glycol mono-salicylate (**7**): 2-hydroxyethyl 2-hydroxybenzoate

A total of 15 g of salicylic acid (109 mmol), 30 ml of ethylene glycol (807 mmol) and concentrated H_2_SO_4_ (3 ml, 57 mmol) were refluxed for 1 hr at 50°C. TLC monitored the reaction using *n*-hexane/ethyl acetate (1/1, v/v). After completion, the reaction mixture was cooled down at room temperature and dissolved in distilled H_2_O. The organic phase was collected, washed with an aqueous solution of NaHCO_3_ (5%) and three times with distilled H_2_O till neutral pH, then dried with anhydrous Na_2_SO_4_. After filtration and evaporation of the organic phase, the crude extract was purified by silica gel column chromatography (hexane/ethyl acetate: 2/1, v/v) to yield monoethylene glycol mono-salicylate (**7**). The chemical structure of **7** was confirmed by analyses of its spectroscopic data (FTIR, NMR and HRMS-ESI).

Clear oily liquid (13.22 g, 67.3%). FTIR (υ_max_ cm^−1^): 3189 (OH), 1671 (conj. C=O), 1614, 1585, 1485 (C=C), 1247 (C–O–C). δ_H_ (500 MHz, CDCl_3_, Me4Si): 10. 68 (1H, s, OH), 7.85 (1H, d, *J* = 7.5 Hz, Ph), 7.47 (1H, td, *J* = 7.0 & 1.5 Hz, Ph), 6.99 (1H, d, *J* = 8.0 Hz, Ph), 6.85 (1H, t, *J* = 7.5 Hz, Ph), 4.55 (2H, t, *J* = 4.5 Hz, COO-C*H*_2_-), 3.91 (2H, t, *J* = 4.5 Hz, C*H*_2_-OH. δ_C_ (125 MHz, CDCl_3_, Me4Si): 170.0, 161.6, 117.6, 135.9, 119.3, 130.0, 112.3, 72.5, 68.9, 64.3, 61.8. HR-MS-ESI in a positive mode (*m/z*): calcd for C_9_H_10_O_4_ [M]^−^ = 182.0584; C_9_H_9_O_4_ [M−H]^−^ = 181.0506. Found [M−H]^−^ = 181.0512.

#### 3.2.2.3. Synthesis of cinnamate-salicylate (**12**) and monoethylene glycol disalicylate (**13**)

Equimolar amounts of ethylene glycol monosalicylate (1 g, 5.49 mmol) and cinnamic acid (0.81 g, 5.49 mmol) were dissolved in toluene in a round bottom flask in the presence of 0.181 g of TsOH (10% of the reagent amounts). The mixture was refluxed for 2 h under azeotrope distillation. The reaction was monitored by TLC and after completion, it was cooled at room temperature, washed three times with distilled water till neutral pH. The organic phase was collected, dried with anhydrous Na_2_SO_4_, filtered and evaporated under vacuum. The dark brown residue obtained was purified by silica gel column chromatography using the solvent mixture *n*-hexane/ethyl acetate (15/1, v/v) to give pure cinnamate-salicylate (**12**) and a polyethylene glycol di-salicylate (**13**). The syntheses of compounds **12** and **13** were established by means of common spectroscopic methods, including FTIR, NMR and HRMS-ESI.

*Cinnamate-salicylate (* ***12*** *): (E)-2-(cinnamoyloxy)ethyl 2-hydroxybenzoate*

Light-yellowish powder (0.60 g, 35.1%). FTIR (υ_max_ cm^−1^): 3220 (OH), 1698, 1670 (C=O), 1609, 1579 (C=C), 1157.53 (C-O-C). *δ*_H_ (500 MHz, CDCl_3_, Me4Si): 10.66 (1H, s, OH), 7.91 (1H, dd, *J* = 8.0 & 1.7 Hz, Ph-cinnamate), 7.76 (1H, d, *J*_trans_ = 16.0 Hz, CH=CH=COO), 7.56 (2H, m, Ph-cinnamate), 7.49 (2H, m, Ph-salicylate); 7.41 (2H, m, Ph-cinnamate), 7.02 (1H, d, *J* = 8.4 Hz, Ph-salicylate), 6.92 (1H, t, *J* = 8.1 Hz, Ph-salicylate), 6.49 (1H, d, *J*_trans_ = 16.0 Hz, CH=CH-COO), 4.66 (2H, m, O-CH_2_-CH_2_-O), 4.59 (2H, m, O-CH_2_-CH_2_-O). δ_C_ (125 MHz, CDCl_3_, Me4Si): 169.9, 166.6, 161.8, 145.7, 135.9, 134.2, 130.5, 130.1, 128.9, 128.2, 119.3, 119.3, 117.7, 117.6, 117.4, 112.2, 63.1, 62.0. HR-MS-ESI in a positive mode (*m/z*): calcd for C_18_H_16_O_5_ [M]^−^ 312.1003; C_18_H_15_O_5_ [M − H]^−^ = 311.0924. Found [M − H]^−^ = 311.0932.

*Ethane-1,2-diyl bis(2-hydroxybenzoate): monoethylene glycol di-salicylate (* ***13*** *)*

Light-pink powder (0.188 g, 11.3%). FTIR (υ_max_ cm^−1^): 3370 (OH), 2950, 16 711 (C=O), 1247 (C-O-C). δ_H_ (500 MHz, CDCl_3_, Me4Si): 10.61 (2H, s, OH), 7.88 (2H, dd, *J* = 8.0, 1.8 Hz, Ph), 7.49 (2H, ddd, *J* = 8.7, 7.1 & 1.8 Hz, Ph), 7.02 (2H, dd, *J* = 8.4 & 1.1 Hz, Ph), 6.91 (2H, t, *J* = 7.6 Hz, Ph), 4.73 (4H, s, O-C*H*_2_-C*H*_2_-O). δ_C_ (125 MHz, CDCl_3_, Me4Si): 169.8, 161.8, 136.1, 130.0, 119.3, 117.7, 112.0, 62.70. HR-MS-ESI in a positive mode (*m/z*): calcd for C_16_H_14_O_6_ [M]^−^ = 302.0796; C_16_H_12_O_6_Na [M+Na−2H]^−^ = 323.0537. Found [M + Na − 2H]^−^ = 323.0552.

#### 3.2.2.4. Synthesis of cinnamic-ibuprofen ester (**14**): 2-((2-(4-isobutylphenyl)propanoyl)oxy)ethyl cinnamate

A total of 1.5 g of Ibuprofen ethylene glycol (5.99 mmol) and 0.89 g of cinnamic acid (5.99 mmol) were refluxed under azeotrope distillation for 2 h in toluene, with TsOH (10% weight of reagents) as catalyst. After the reaction was completed, the reagent mixture was cooled down and washed several times with distilled H_2_O till pH = 7. The organic phase was collected, dried over anhydrous Na_2_SO_4_, filtered, and the solvent evaporated. The crude extract was purified using silica gel column chromatography with *n*-hexane/ethyl acetate (15/1, v/v) to yield a pure compound, designated as **14**. The chemical structure of this compound was confirmed through FTIR, NMR and HRMS-ESI analyses.

Yellow paste (1.20 g, 70.2%). FTIR (υ_max_ cm^−1^): 2962 (CH), 1734 & 1720 (C=O), 1614, 1077 (C-O-C). *δ*_H_ (500 MHz, CDCl_3_, Me4Si): 7.69 (1H, d, *J*_trans_ = 16.0 Hz, CH=C*H*), 7.55 (2H, m, Ph-cinnamate), 7.42 (3H, m, Ph-cinnamate), 7.23 (2H, m, Ph-ibuprofen), 7.10 (2H, m, Ph-ibuprofen), 6.41 (1H, d, *J*_trans_ = 16.0 Hz, C*H*=CH), 4.39 (4H, m, O-C*H*_2_-C*H*_2_-O), 3.76 (1H, q, *J* = 7.4 Hz, -C*H*-C=O), 2.41 (2H, d, 7.2 Hz, C*H*_2_-CH(CH_3_)_2_), 1.85 (1H, m, (CH_3_)_2_C*H*-CH_2_), 1.52 (3H, d, *J* = 7.5 Hz, C*H*_3_-CH), 0.88 (d, *J* = 6.7 Hz, 6H, (CH_3_)_2_CH_2_). *δ*_C_ (125 MHz, CDCl_3_, Me4Si): 174.5, 166.6, 145.3, 140.6, 137.5, 130.4, 129.3, 128.9, 128.1, 127.1, 117.6, 62.4, 62.2, 45.0, 30.1, 29.6, 22.4, 18.4. HR-MS-ESI in a positive mode (*m/z*): calcd for C_24_H_28_O_4_ [M]^−^ = 380.1993; C_24_H_27_O_4_ [M − H]^−^ = 379.1915. Found [M − H]^−^ = 379.1935.

### 3.2.3. Synthesis of salicylate monoethylene glycol fatty acid esters (DEW4)

Fatty acid esters of monoethylene glycol salicylate (**5**) are compounds that have been synthesized and described previously [30]. The NMR and HRMS spectra are presented in the electronic supplementary material, figures S18–S29.

### 3.2.4. Cytotoxicity and anti-inflammatory studies

#### 3.2.4.1. Preparation of cell culture

Peritoneal macrophages were isolated from four eight-week-old, 25 g Wistar mice by intraperitoneal injection of 0.5 ml of 2% starch solution (inflammatory agent) to stimulate their production [38]. Four days later, the animals were sacrificed, and 5 ml of phosphate buffered saline (PBS) buffer (0.1 M, pH 7.4) was injected into their peritoneal cavity with a syringe. After massage of the peritoneal cavity, the injected buffer was slowly aspirated through a small abdominal incision. The resulting solution containing macrophages was transferred to 15 ml Falcon tubes and kept in ice. The fluid obtained was then centrifuged (1620 g, 4°C and 10 min), and the supernatant was removed. The erythrocytes were removed by osmotic shock. The cells were suspended in 1 ml hypotonic 0.05 M NaCl solution for 1 min. Isotonicity was then restored by adding 1 ml of 0.25 M NaCl [39]. The mixture was centrifuged (1620 g, 4°C and 10 min) and the resulting pellet was suspended in 2 ml of Dulbecco's modified eagle medium (DMEM) (containing 0.2% streptomycin and penicillin, 0.4 g l^−1^ bovine serum albumin and 3.7 g l^−1^ NaHCO_3_ in PBS and filtered with 0.22 µm Millipore filters) [40].

Cell viability was determined by the trypan blue exclusion method, which is based on the principle that living cells have intact membranes that exclude certain dyes (trypan blue, eosin or propidium), whereas dead cells do not. Cell viability is calculated as the number of viable cells divided by the total number of cells within the grids on the haemocytometer. If cells absorb trypan blue, they are considered non-viable. A 0.4% solution of trypan blue in PBS was prepared, 0.1 ml of this solution was added to 0.1 ml of cells and the resulting solution was loaded into a haemocytometer and immediately examined under a microscope at low magnification. The number of blue-stained cells (dead cells) and the total number of cells were then counted. The cell viability was calculated as follows:


$$\%\;Cell\;Viability=\frac{Total\;Cells-Dead\;Cells}{Total\;Cells}\times 100.$$

#### 3.2.4.2. Treatment of isolated macrophages with tested samples

In the 96-well plate, 150 µl of a cell suspension (104 cells per well) were distributed in different wells. In the test and positive control wells, 50 µl of *Saccharomycces cerevisiae* (250 µg ml^–1^) was added to stimulate the production of pro-inflammatory cytokines. Meanwhile 50 µl of DMEM was added in the negative control wells. The microplate was incubated at 37°C (5% CO_2_) for 1 h, then 50 µl of solution of compounds or baicalin at different concentrations (0.241 × 10^–3^, 2.23 × 10^–3^, 22.4 × 10^–3^, 220 × 10^–3^ and 2410 × 10^–3^ mM) were added to the test wells and 50 µl DMEM to the positive (cells activated with *Saccharomyces cerevisiae*) and negative (inactivated cells) control wells. After 3 h of incubation at 37°C (5% CO_2_), the cell supernatants were used for NO [41].

#### 3.2.4.3. Cell viability assay

The effect of the solutions of synthetic compounds on the viability of macrophages in primary culture was determined by using the MTT as previously described in the literature [42]. The cell pellet from the different incubations was taken up in 100 µl of MTT solution (0.5 mg ml^−1^ in PBS) and the mixture was incubated at 37°C for 90 min, then the supernatant was removed and 100 µl of acidified isopropanol was added to each tube to dissolve the formazan crystals formed. Finally, the absorbance of the purple solution was read at 630 nm against the acidified isopropanol solution. The percentage of cell viability was calculated using the following formula:


$$\%viability=\left(OD_{assay}/OD_{control}\times 100\right).$$

#### 3.2.4.4. Quantification of nitric oxide production by stimulated macrophages

A total of 100 µl of the previously obtained cell supernatants were mixed with 100 µl of Griess reagent (1% sulphanylamide, 0.1% naphthyl ethylenediamine dihydrochloride in 2.5% v/v phosphoric acid). The mixture was incubated for 10 min at room temperature and the absorbance is read at 550 nm. The amount of nitrite was measured using the standard sodium nitrate curve [43]. The percentage inhibition of nitric oxide synthesis was calculated using the following formula:


%inhibition=[(ODcontrol−ODassay/ODcontrol]×100).

#### 3.2.4.5. Statistical analysis

All experiments were performed in triplicate. Results are expressed as mean ± s.d. The normal distribution of data was verified using the Shapiro–Wilk test. Statistical significance was determined by analysis of variance (ANOVA) using Graph Pad Prism 8.0.1 for Windows, followed by a Tukey post hoc test. For statistical analysis, significance levels were set at *p* ˂ 0.0001.

### 3.2.5. Antioxidative studies

#### 3.2.5.1. Scavenging 2,2-diphenyl−1-picrylhydrazyl radicals

The DPPH radical trapping protocol is based on Bassene’s method [35], modified for colorimetric analysis. DPPH is a stable purple organic nitrogen radical that absorbs light at 517 nm. When an anti-free radical agent is present, DPPH is reduced to a stable yellow compound, indicated by a loss of colour between 470 and 517 nm. A 0.02% DPPH solution was made with ethanol and stored in a sealed jar in the dark. Stock solutions of the synthesized compounds were prepared by dissolving 100 mg of each in 1 ml of DMSO, and the final concentration of DMSO concentration in the first well was 0.5%. These stocks were then diluted to achieve concentrations (500, 250, 125, 62.5, 31.25, 15.625 and 7.8125)/Mi in mM (Mi: molecular weight of each synthetic compound). Each dilution (25 µl) was put to the wells and 75 µl of the DPPH solution (0.02%) were added. Optical densities were measured at 517 nm after 30 min of incubation in the dark. The negative control used DPPH alone, while the positive control used gallic acid treated with solutions of the synthesized compounds at final concentrations (50, 25, 12.5, 6.25, 3.125, 1.5625 and 0.78125)/Mi in mM. Synthesized compounds were also tested alone to assess fluorescence.

#### 3.2.5.2. Scavenging 2,2′-azino-bis(3-ethylbenzothiazoline-6-sulfonate) radicals

The modified technique by Khan *et al.* [34] was used to scavenge ABTS^+•^ cation radicals through a discoloration test. In this test, potassium permanganate (KMnO_4_) or potassium persulphate (K_2_S_2_O_8_) reacts with ABTS to generate a blue-green ABTS^+•^ radical. Antioxidants reduce this radical, leading to a measurable colour change at 734 nm, proportional to the antioxidant concentration. The antioxidant capacity is assessed by comparing the intensity reduction of ABTS colour with that of a reference antioxidant, gallic acid. Stock and dilute solutions of synthetic compounds were prepared as detailed in §3.2.5.1. Diluted concentrations were (50, 25, 12.5, 6.25, 3.125, 1.5625 and 0.78125)/Mi in mM. The ABTS solution was obtained by mixing ABTS with K_2_S_2_O_8_, producing an intense blue colour. The ABTS^+•^ radical was generated by combining equal volumes of a 4.9 mM K_2_S_2_O_8_ solution and a 7 mM ABTS stock solution, stored in the dark at room temperature for 15 h before use. The 7 mM ABTS stock was then diluted 20 times with distilled water. A total of 25 µl of each dilution was added to wells along with 75 µl of 0.175 mM ABTS+ solution. After 30 min of incubation in the dark at room temperature, optical densities were measured at 734 nm [44]. Negative controls contained the ABTS reagent without synthesized compounds, while positive controls used gallic acid at the same final concentrations as the compounds. Compounds were also tested alone to assess fluorescence. The reduction of ABTS results in a blue discoloration, with lower absorbance indicating higher antioxidant activity [45].

#### 3.2.5.3. Ferric reducing antioxidant power test

The Fe^3+^ reduction test followed Path Canada’s protocol [36] with some modifications, assessing the reduction of Fe^3+^ ions to Fe^2+^ ions, which form a red-orange complex with 1,10-phenanthroline, measurable at 505 nm. The colour intensity correlates with the amount of Fe^3+^ reduced. Four solutions were prepared: 1.2 mg ml^−1^ of Fe^3+^ solution (1.2 mg FeCl_3_ in 1 ml distilled H_2_O), 0.2% ortho-phenanthroline solution (200 mg powder in 100 ml ethanol), 2 mg ml^−1^ of gallic acid solution (2 mg gallic acid in 1 ml distilled H_2_O) as a positive control, stock and dilute solutions of the synthesized compounds (see §3.2.5.1). In a 96-well microplate, 180 µl distilled H_2_O was added to the first column and 100 µl to others for dilution. Each compound solution (20 µl at 10 mg ml^−1^) was mixed with 25 µl Fe^3+^ solution and incubated for 15 min in the dark at room temperature. Afterwards, 50 µl ortho-phenanthroline solution was added, followed by another 15 min incubation. Optical density was measured at 505 nm using a TECAN M200 plate reader. The positive control was treated similarly to the DPPH and ABTS assays.

#### 3.2.5.4. Results expression

Assays were performed in duplicate, and results were averaged. Percentage inhibition (PI), reflecting the antioxidant activity related to the DPPH˙ radical scavenging effect, was calculated using the following formula:


$$\left(PI\right)=\frac{Acontrol-Atest}{Acontrol}\times 100,$$

where Acontrol = absorbance of the control sample and Atest = absorbance of the tested sample.

The percentages of scavenging or 50% reduction were calculated using gallic acid as a standard for 100% reduction. The concentration of radical scavengers needed to neutralize 50% of free radicals, referred to as SC_50_, was determined using GraphPad Prism 8.0.1 software (244).

#### 3.2.5.5. Statistical analysis

All the experiments were performed in duplicate. The scavenging concentrations of 50% radicals were reported as the mean ± s.d. Statistical analysis was conducted using ANOVA, followed by Dunnett’s test for post hoc comparisons. The test was considered to have a significant difference at *p* ≤ 0.05.

## 4. Conclusion

In an effort to develop multifunctional drugs that alleviate the burden of patients suffering from multiple inflammation-related diseases. Several compounds derived from anti-inflammatory drugs were synthesized and characterized by common spectroscopic method, six were identified as new ones. Cytotoxicity and anti-inflammatory properties were evaluated using the MTT assay on mouse-derived peritoneal macrophages. The results showed that all tested compounds were safe up to 50% concentrations (2.41 × 10^−^⁴ to 2.41 mM), with monoethylene glycol di-ibuprofen (**2**) exhibiting the highest toxicity due to its high lipophilicity. Most compounds demonstrated non-toxicity below 2.41 mM and concentration-dependent inhibition of NO production at 0.24 mM. Particularly, some synthesized compounds displayed superior anti-inflammatory activity compared with ibuprofen, with monoethylene glycol mono-ibuprofen (**3**) showing the most potent inhibitory activity (IC_50_ = 0.002 ± 0.00 mM). Structure–activity relationship analysis revealed that primary alcoholic and mono-ester groups enhanced anti-inflammatory properties, while phenolic OH, di-ester groups, and long aliphatic chains decreased anti-inflammatory activity. Furthermore, antioxidant activity assays (DPPH, FRAP and ABTS) revealed that the synthesized esters exhibited limited antioxidant capabilities. Notably, only a few compounds demonstrated modest activity in reducing the ABTS+ radical, indicating a weak antioxidant potential. These findings suggest that the synthesized compounds may not be effective antioxidants, but their anti-inflammatory properties make them potential candidates for further development as anti-inflammatory or anti-cancer agents.

## Data Availability

The datasets, including the spectra used for the structural confirmation of the esters synthesized and described in this manuscript, are available as electronic supplementary material at Dryad [46]. Supplementary material is available online [47].
